# Small vessel disease burden predicts functional outcomes in patients with acute ischemic stroke using machine learning

**DOI:** 10.1111/cns.14071

**Published:** 2023-01-17

**Authors:** Xueyang Wang, Jinhao Lyu, Zhihua Meng, Xiaoyan Wu, Wen Chen, Guohua Wang, Qingliang Niu, Xin Li, Yitong Bian, Dan Han, Weiting Guo, Shuai Yang, Xiangbing Bian, Yina Lan, Liuxian Wang, Qi Duan, Tingyang Zhang, Caohui Duan, Chenglin Tian, Ling Chen, Xin Lou, the MR‐STARS Investigators

**Affiliations:** ^1^ Medical School of Chinese PLA Beijing China; ^2^ Department of Radiology Chinese PLA General Hospital Beijing China; ^3^ Department of Radiology Yuebei People's Hospital Guangdong China; ^4^ Department of Radiology Anshan Changda Hospital Liaoning China; ^5^ Department of Radiology Shiyan Taihe Hospital Hubei China; ^6^ Department of Radiology Qingdao Municipal Hospital Affiliated to Qingdao University Qingdao China; ^7^ Department of Radiology WeiFang Traditional Chinese Hospital Shandong China; ^8^ Department of Radiology The Second Hospital of Jilin university Jilin China; ^9^ Department of Radiology The First Affiliated Hospital of Xi'an Jiaotong University Shaanxi China; ^10^ Department of Radiology The First Affiliated Hospital of Kunming Medical University Yunnan China; ^11^ Department of Radiology Shanxi Provincial People's Hospital Shanxi China; ^12^ Department of Radiology Xiangya Hospital Central South University Hunan China; ^13^ Department of Neurology Chinese PLA General Hospital Beijing China; ^14^ Department of Neurosurgery Chinese PLA General Hospital Beijing China

**Keywords:** acute ischemic stroke, cerebral small vessel disease, machine learning, prediction model

## Abstract

**Aims:**

Our purpose is to assess the role of cerebral small vessel disease (SVD) in prediction models in patients with different subtypes of acute ischemic stroke (AIS).

**Methods:**

We enrolled 398 small‐vessel occlusion (SVO) and 175 large artery atherosclerosis (LAA) AIS patients. Functional outcomes were assessed using the modified Rankin Scale (mRS) at 90 days. MRI was performed to assess white matter hyperintensity (WMH), perivascular space (PVS), lacune, and cerebral microbleed (CMB). Logistic regression (LR) and machine learning (ML) were used to develop predictive models to assess the influences of SVD on the prognosis.

**Results:**

In the feature evaluation of SVO‐AIS for different outcomes, the modified total SVD score (Gain: 0.38, 0.28) has the maximum weight, and periventricular WMH (Gain: 0.07, 0.09) was more important than deep WMH (Gain: 0.01, 0.01) in prognosis. In SVO‐AIS, SVD performed better than regular clinical data, which is the opposite of LAA‐AIS. Among all models, eXtreme gradient boosting (XGBoost) method with optimal index (OI) has the best performance to predict excellent outcome in SVO‐AIS. [0.91 (0.84–0.97)].

**Conclusions:**

Our results revealed that different SVD markers had distinct prognostic weights in AIS patients, and SVD burden alone may accurately predict the SVO‐AIS patients' prognosis.

## Graphical abstract

Characteristics of cerebral small vessel disease (CSVD) in acute ischemic stroke (AIS) patients can affect outcomes at 90 days. Meanwhile, different imaging markers of CSVD have different weights of impact on large artery atherosclerosis and small vessel occlusion subtype AIS.

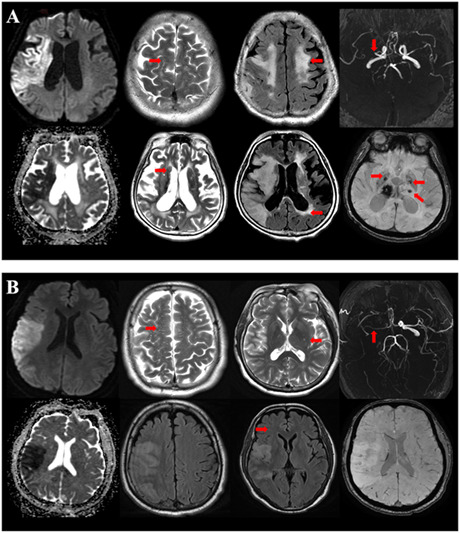

## 1 INTRODUCTION

A distinct burden of cerebral small vessel disease (SVD) imaging markers is frequently detected in acute ischemic stroke (AIS) patients. The imaging features of SVD on MRI include chronic lacunes, cerebral microbleeds (CMB), white matter hyperintensity (WMH), and perivascular space (PVS).^1^ Although cerebral SVD imaging markers have been shown to be associated with an increased risk of stroke recurrence, death, disability, and worse clinical outcome, it is unknown whether SVD burden could increase the predictive power of different types of AIS prognosis.^2^, ^3^, ^4^

Previously, for AIS patients, the focus was limited to clinical factors, such as age, stroke severity, and time from stroke onset, in the prediction of prognosis.^5^ Furthermore, information from multimodal magnetic resonance imaging (MRI), such as the use of the arterial spin label (ASL) sequence to quantify collateral perfusion, can also be used to better understand the prognosis of ischemic stroke patients.^6^ However, SVD burden such as WMH and lacunes, which, may be identified promptly and clearly on standard MRI, are frequently neglected in the examination of AIS. Few studies have been conducted to determine prognosis based on SVD burden. In addition, most traditional research employed the regular logistical regression (LR) approach to quantify correlations in terms of odds ratios (ORs), which does not necessarily mean that the markers would be able to reliably discriminate patients with different outcomes. In contrast, machine learning (ML) methods have various algorithms that do not require the linear assumption and can also control collinearity with regularized hyperparameters.^7^ So innovative combination of SVD imaging markers and clinical predictors using different ML algorithms such as random forest (RF) and eXtreme Gradient Boosting (XGBoost) to build predictive models for different types of AIS is essential.

Thus, the purpose of the present study was to investigate the predictive efficacy of total SVD burden for functional outcome at 90 days in AIS patients with small‐vessel occlusion (SVO) and large‐artery atherosclerosis (LAA) types, which are commonly encountered in clinical practice. We hypothesized that SVD‐related imaging markers may increase prognostic information, especially in SVO‐AIS patients, whether using LR or ML approaches.

## 2 MATERIALS AND METHODS

### 2.1 Patients

We retrospectively evaluated patients from two cohorts: a multicenter registry (MR‐STARS, Clinicaltrials. gov, NCT02580097) from December 2018 to March 2021 and the local hospital's previous database. MR‐STARS is population‐based study of all stroke events occurring within 24 h, consisted of 961 individuals. Local hospital's previous database included 650 consecutive cases of TIA/ischemic stroke recruited from October 2015 to December 2018. Two clinically common subtypes (SVO and LAA) of acute anterior circulation ischemic stroke were included in this study, both receiving the same standard conservative medical treatment. This research was approved by ethics committee authorities in all participating groups, and written informed consent was obtained from patients. This research was performed in accordance with the Declaration of Helsinki. Patients provided written consent. MR‐STARS was registered (S2018‐193‐01) and approved by ethics committee authorities in all participating groups.

### 2.2 Collection of demographic and clinical data

Demographic and clinical data were collected through a multicenter and local hospital dataset. The following stroke risk factors were identified: age, sex, hypertension, hyperglycemia, hyperlipidemia, coronary heart disease, atrial fibrillation, smoking, and alcohol consumption. Baseline characteristics, including onset to admission, National Institutes of Health Stroke Scale (NIHSS) score, mRS score, and previous medication use, were also assessed at the time of initial presentation as part of the admission workup.

### 2.3 Outcome measures

Clinical follow‐up of AIS patients was performed by trained raters (Z.H.M., X.Y.W., W.C., G.H.W., Q.L.N., X.L., Y.T.B., D.H., W.T.G., and S.Y.) blinded to the imaging data using the mRS 3 months after stroke via a telephone interview or outpatient follow‐up and defined as follows: 1. Excellent outcome (mRS 0–1); 2. Good outcome (mRS 0–2).

### 2.4 Brain MRI acquisition

Before treatment, multimodal MRI was performed as soon as possible after admission using a 3.0 T scanner (GE Discovery 750 W) with a 32‐channel head coil using standardized protocols. The imaging protocol included three‐dimensional time‐of‐flight magnetic resonance angiography (3D TOF‐MRA), diffusion‐weighted imaging (DWI), T1‐weighted fast spin echo (T1W FSE), T2‐weighted FSE (T2W FSE), T2 fluid attenuated inversion recovery (T2 FLAIR), and susceptibility‐weighted imaging (SWI).

### 2.5 Classification of stroke types

Stroke events were defined according to the World Health Organization criteria, and all cases were confirmed using neuroimaging. Stroke subtypes were divided as LAA, SVO, cardioembolic (CE), other causes, and undetermined according to the Trial of Org 10,172 in Acute Stroke Treatment (TOAST) classification criteria, subsequently LAA and SVO AIS were included for analysis.^8^

LAA‐AIS were defined as patients with clinical or brain imaging findings of either significant stenosis or occlusion of the middle or anterior cerebral artery and presumably due to atherosclerosis based on MRA sequence.

SVO‐AIS were identified as occlusion of the small perforating arteries deep in the cerebral hemisphere, unaccompanied by stenosis or occlusion of the main trunk of the intracranial large arteries in the MRA sequence, with lesions ranging in size from 2 to 20 mm, as demonstrated in the DWI sequence.^8^, ^9^

### 2.6 Imaging assessment

The infarction lesion volume was measured manually using NeuBrainCARE software by tracing the margin of the hyperintense lesion on each lesion slice on the DWI map corresponding to apparent diffusion coefficient (ADC) maps and computing the overall volume.

The SVD burden was assessed by two radiologists (X.Y.W. and Q.D.) who were blinded to the clinical history, patient identity, and prognosis of the patients. Any disparity was resolved by a senior radiologist (J.H.L.). The identification of SVD imaging markers, such as chronic lacune, CMB, WMH, and PVS, was based on the STandards for ReportIng Vascular changes on nEuroimaging (STRIVE).^1^ The severity of WMH was assessed according to the Fazekas scoring system.^10^ The centrum semiovale PVS (CS‐PVS) and basal ganglia PVS (BG‐PVS) were assessed with a validated four‐point visual rating scale (0 = none; 1 = 1–10; 2 = 11–20; 3 = 21–40; and 4= > 40).^11^

To calculate the total SVD score, we evaluated chronic lacune, CMB, WMH, and PVS based on the ordinal scale from 0 to 4, which has been widely used by several studies.^12^, ^13^ The modified total SVD score (0–6) was also applied in the present study to assess the following: presence of lacunes, 1 point; 1–4 CMBs, 1 point; ≥5 CMBs, 2 points; >20 BG‐EPVS, 1 point; moderate WMH (total Fazekas = 3–4), 1 point; and severe WMH (total Fazekas = 5–6), 2 points^14^ (Table S1).

### 2.7 LR prediction model development

LR models were established in LAA and SVO AIS patients, and each index was screened using the bidirectional stepwise regression method. Classify all the indexes according to their characteristics as: regular index (RI), which included sex, age, onset to admission (OTA) time, NIHSS score, and history of smoke, alcohol, chronic heart disease, atrial fibrillation, hypertension, hyperlipidemia, hyperglycemia, premedication, aspirin, clopidogrel, statin; image index (II), which mainly contained SVD burden, such as the scores of deep‐WMH (D‐WMH), periventricular‐WMH (P‐WMH), CS‐PVS, BG‐PVS, and presences of lacune, CMB, total SVD score and modified total SVD score were similarly included in the II input, while in LAA‐AIS population, the quantity of infarcts on DWI images is also integrated into II input; total index (TI), RI and II together. These different combinations of indexes were entered to assess the predictive power of different models. The LR prediction models were established in R software. (Version 4.0.4).

### 2.8 ML prediction model development

As no one technique is accurate in every field and ML methods in different research areas and data sets may yield different results, so we adopted 3 ML algorithms in models: Gaussian process regression (GPR), RF, and XGBoost.

GPR algorithm is a nonparametric Bayesian regression method that has an excellent performance on small data sets, and with no model parameters that need to be set subjectively. RF method builds multiple decision trees through random sampling with replacement, and the output results of multiple decision trees are voted to predict which category they belong to. For the XGBoost model, it has optimized the decision tree algorithm to improve the handling of the data set. The accuracy is enhanced through regularization and built‐in cross‐validation to address the overfitting problem. In the RF and XGBoost algorithms, parameter calibrations were built using the grasshopper optimization algorithm (GOA) method, which ensured that the algorithm has a strong global search capability and can effectively avoid stagnation in the local optimum.

Indexes classification in ML methods was similar to LR models, distinguished as RI, II, TI, and built different prediction models. In addition, importance ranking of all features was performed in the XGBoost model. The XGBoost model usually determines the importance of a tree by three indicators, namely Gain, Frequency, and Cover. Gain expresses the importance of the dendritic feature, and a higher value of this metric means that it is more important for generating predictions; Frequency indicates the relative percentage of times a particular feature occurs in the model tree. Cover refers to the relative number of observations associated with this function. Gain is the most relevant attribute that explains the relative importance of each feature. In the present research, when the gain value of the index was above its threshold (>0.01), it was classified as an optimal index (OI) and also introduced into the ML models like RI, II, and TI. ML models were established in R software (Version 4.0.4).

### 2.9 Model performance

After the models were derived, the average receiver operator characteristics (ROC) curves and mean area under the ROC curve (AUC) with 95% confidence intervals (CI) for all models were also calculated. An internal validation was used through a repeated fivefold cross‐validation procedure to correct the AUC of each model for optimism. The performances of different models were also compared by ROC analysis and using the Delong test. ROC curves and AUC values were computed using R software. (Version 4.0.4).

### 2.10 Statistical Analysis

Continuous variables were expressed as the mean ± SD or median with interquartile range depending on the distribution of the variable. Normality of the distributions was assessed by the Shapiro–Wilk test. In univariate analyses, normally distributed continuous variables were compared with Student's t test, and the variables not normally distributed were compared with the Kruskal–Wallis H test or Mann–Whitney *U* test. Categorical variables were instead presented as percentages and were compared with Pearson's chi‐square test or Fisher's exact test. The simple Cohen kappa statistic was used for the assessment of different SVD imaging markers. All statistical analyses were performed using SPSS version 26.0. (IBM Corporation).

## 3 RESULTS

### 3.1 Patient characteristics

A total of 398 SVO‐AIS patients and 175 LAA‐AIS patients were included separately in this study for analysis with less than 24 h from onset to admission time and received the same standard conservative medication (Figure S1). The median age of the SVO‐AIS patients was 63.0 ± 18.9 years and 74.3% were men. Similarly, LAA‐AIS patients was 63.5 ± 13.9 years and 70.3% were men.

#### 3.1.1 Functional outcome

In SVO‐AIS population, the proportions of patients with excellent and good outcomes were 60% (239/398) and 75.4% (300/398). In LAA‐AIS patients, the proportions of patients with excellent and good outcomes were 34.9% (61/175) and 52% (91/175), respectively, respectively.

The clinical and imaging characteristics of functional outcomes in different subtypes AIS patients are presented in Tables 1 and 2. Both SVO and LAA AIS patients with excellent or good outcomes were younger and had a lower baseline NIHSS score and a lower SVD burden.

**TABLE 1:** Clinical and imaging characteristics of the study population based on functional outcomes in SVO‐AIS patients.

	Functional outcome					
	mRS (0–1) *n* = 239	mRS (2–6) *n* = 159	*p*‐Value	mRS (0–2) *n* = 300	mRS (3–6) *n* = 98	*p*‐Value
Demographics						
Sex, male, *n* (%)	187 (78.2)	109 (68.6)	0.03	233 (77.7)	63 (64.3)	0.008
Age, years, mean ± SD	58.9 ± 11.1	69.2 ± 25.4	<0.01	59.9 ± 11.3	72.7 ± 30.8	<0.01
Clinical history *n* (%)						
Hypertension	150 (62.8)	116 (73)	0.034	193 (64.3)	73 (74.5)	0.064
Hyperglycemia	85 (35.6)	77 (48.4)	0.011	110 (36.7)	52 (53.1)	0.004
Hyperlipemia	122 (51)	61 (38.4)	0.013	142 (47.3)	41 (41.8)	0.343
CHD	29 (12.1)	30 (18.9)	0.064	43 (14.3)	16 (16.3)	0.63
AF	12 (5.0)	13 (8.2)	0.204	15 (5)	10 (10.2)	0.065
Smoke	112 (46.9)	59 (37.1)	0.054	137 (45.7)	34 (34.7)	0.057
Alcohol	76 (31.8)	42 (26.4)	0.249	95 (31.7)	23 (23.5)	0.123
Statin	54 (22.6)	54 (34)	0.012	72 (24)	36 (36.7)	0.014
Aspirin	53 (22.2)	51 (32.1)	0.028	71 (23.7)	33 (33.7)	0.05
Clopidogre	44 (18.4)	39 (24.5)	0.141	58 (19.3)	25 (25.5)	0.191
Premedication	58 (24.3)	60 (37.7)	0.004	79 (26.3)	39 (39.8)	0.011
Clinical variables						
NIHSS, ml (IQR)	2 (1–4)	5 (3–8)	<0.01	3 (2–4)	6 (4–9)	<0.01
Onset‐admission, minute (IQR)	390 (238–634)	480 (280–840)	0.028	416 (240–656)	480 (284–859)	0.066
SVD						
P‐WMH, median (IQR)	1 (1–1)	2 (2–3)	<0.01	1 (1–2)	3 (2–3)	<0.01
D‐WMH, median (IQR)	1 (0–1)	2 (1–3)	<0.01	1 (1–1)	3 (2–3)	<0.01
CS‐PVS, median (IQR)	2 (1–3)	2 (2–3)	0.002	2 (1–3)	2 (2–3)	0.008
BG‐PVS, median (IQR)	1 (1–2)	4 (2–4)	<0.01	1 (1–2)	4 (3–4)	<0.01
CMB, *n* (%)						
0 (0)	206 (86.2)	63 (39.6)	<0.01	240 (80)	29 (29.6)	<0.01
1 (<5)	33 (13.8)	59 (37.1)		57 (19)	35 (35.7)	
2 (≥5)	0	37 (23.3)		3 (1)	34 (34.7)	
Lacune, *n* (%)	54 (22.6)	117 (73.6)	<0.01	85 (28.3)	86 (87.8)	<0.01
Total SVD score, median (IQR)	1 (0–2)	3 (2–4)	<0.01	1 (0–2)	4 (3–4)	<0.01
Modified total SVD score, median (IQR)	0 (0–1)	4 (2–5)	<0.01	1 (0–2)	5 (3–6)	<0.01

Abbreviations: AF, atrial fibrillation; AIS, acute ischemic stroke; BG‐PVS, basal ganglia perivascular space; CHD, coronary heart disease; CMB, cerebral microbleed; CS‐PVS, central semiovale perivascular space; D‐WMH, deep white matter hyperintensity; IQR, interquartile range; mRS, modified Ranking scale; NIHSS, National Institute of Health Stroke Scale; P‐WMH, periventricular white matter hyperintensity; SVD, small vessel disease; SVO, small vessel occlusion.

**TABLE 2:** Clinical and imaging characteristics of the study population based on functional outcomes in LAA‐AIS patients.

	Functional outcome					
	mRS (0–1) *n* = 61	mRS (2–6) *n* = 114	*p*‐Value	mRS (0–2) *n* = 91	mRS (3–6) *n* = 84	*p*‐Value
Demographics						
Sex, male, *n* (%)	45 (73.8)	78 (68.4)	0.461	72 (79.1)	51 (60.7)	0.008
Age, years, mean ± SD	59.3 ± 14.8	65.8 ± 12.9	0.003	60.0 ± 14.54	67.3 ± 12.2	<0.01
Clinical history *n* (%)						
Hypertension	35 (57.4)	77 (67.5)	0.182	56 (61.5)	56 (66.7)	0.480
Hyperglycemia	26 (42.6)	38 (33.3)	0.224	34 (37.4)	30 (35.7)	0.821
Hyperlipemia	15 (24.6)	34 (29.8)	0.893	25 (27.5)	24 (28.6)	0.871
CHD	11 (18.0)	23 (20.2)	0.733	16 (17.6)	18 (21.4)	0.521
AF	4 (6.6)	18 (15.8)	0.079	8 (8.8)	14 (16.7)	0.116
Smoke	35 (42.6)	64 (43.9)	0.875	37 (40.7)	39 (46.4)	0.442
Alcohol	19 (31.1)	27 (23.7)	0.285	26 (28.6)	20 (23.8)	0.475
Statin	21 (34.4)	46 (40.4)	0.442	36 (39.6)	31 (36.9)	0.718
Aspirin	20 (32.8)	51 (44.7)	0.125	41 (45.1)	30 (35.7)	0.209
Clopidogre	16 (26.2)	30 (26.3)	0.990	27 (29.7)	19 (22.6)	0.290
Premedication	22 (36.1)	54 (47.4)	0.151	43 (43.3)	33 (39.3)	0.288
Clinical variables						
DWI‐Quantity, ml, median (IQR)	2.4 (0.3–9.3)	14.3 (2.8–49.4)	<0.01	2.8 (0.4–10.5)	19.2 (6.2–69.2)	<0.01
NIHSS score, median (IQR)	3 (2–4.5)	9 (5.75–12.25)	<0.01	4 (2–8)	10 (6–13)	<0.01
Onset‐admission time, minute (IQR)	484 (252–1258)	420 (305–984)	0.423	484 (311–1201)	420 (246–987)	0.336
SVD						
P‐WMH, median (IQR)	1 (1–2)	2 (1–3)	<0.01	1 (1–2)	2 (1–3)	<0.01
D‐WMH, median (IQR)	1 (1–1)	2 (1–3)	<0.01	1 (1–2)	2 (1–3)	<0.01
CS‐PVS, median (IQR)	2 (1–3)	3 (2–3)	0.016	2 (1–3)	2 (2–3)	0.238
BG‐PVS, median (IQR)	1 (1–2)	2 (1–3)	<0.01	1 (1–2)	3 (2–4)	<0.01
CMB, *n* (%)						
0 (0)	52 (85.2)	66 (57.9)	0.001	68 (74.7)	50 (59.5)	0.094
1 (<5)	6 (9.8)	40 (35.1)		18 (19.8)	28 (33.3)	
2 (≥5)	3 (4.9)	8 (7.0)		5 (5.5)	6 (7.1)	
Lacune, *n* (%)	13 (21.3)	66 (57.9)	<0.01	26 (28.6)	53 (63.1)	<0.01
Total SVD score, median (IQR)	0 (0–1)	3 (1–3.25)	<0.01	1 (0–2)	3 (1–4)	<0.01
Modified total SVD score, median (IQR)	0 (0–1.5)	3 (1–4)	<0.01	1 (0–2)	3 (2–4)	<0.01

Abbreviations: AF, atrial fibrillation; AIS, acute ischemic stroke; BG‐PVS, basal ganglia perivascular space; CHD, coronary heart disease; CMB, cerebral microbleed; CS‐PVS, central semiovale perivascular space; DWI, diffusion weight image; D‐WMH, deep white matter hyperintensity; IQR, interquartile range; LAA, large artery atherosclerosis; mRS, modified Ranking scale; NIHSS, National Institute of Health Stroke Scale; P‐WMH, periventricular white matter hyperintensity; SVD, small vessel disease.

### 3.2 Model performance

**FIGURE 1:**
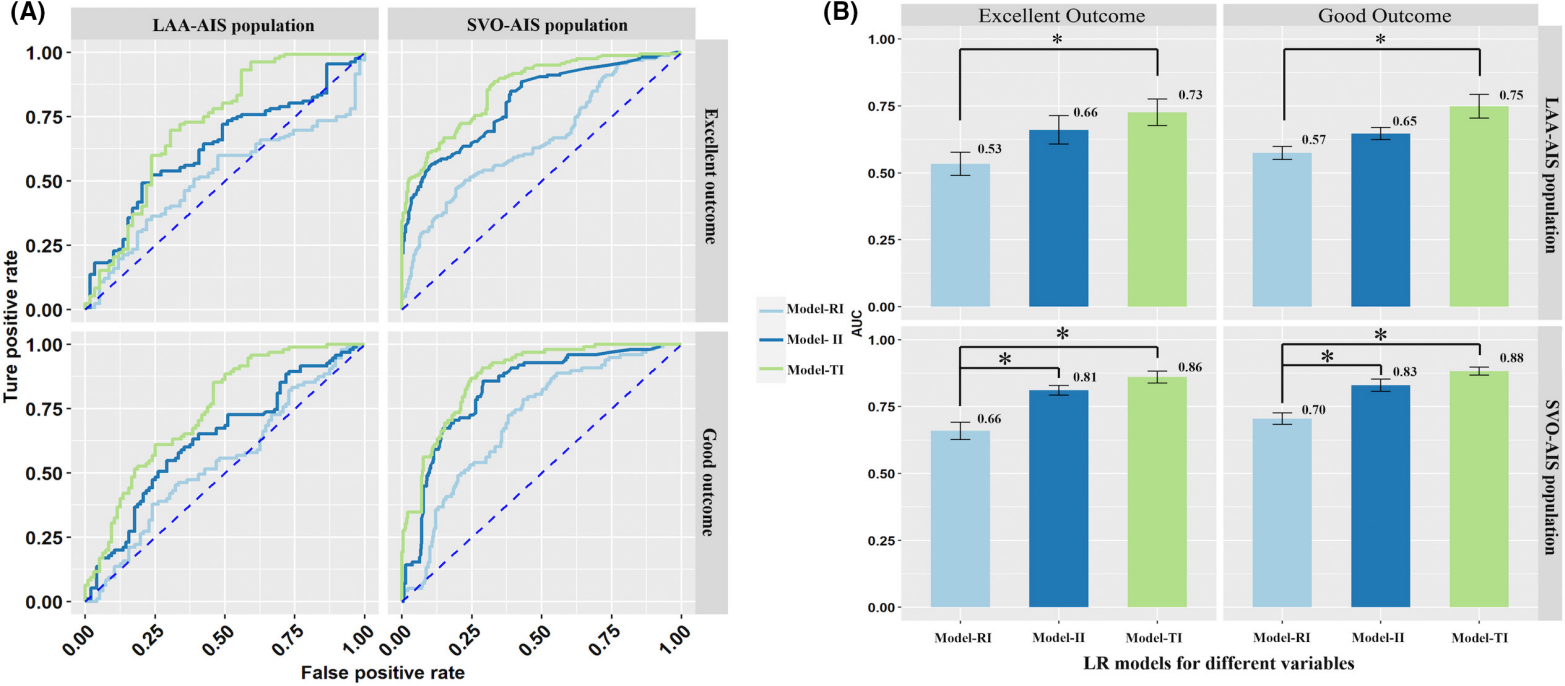
The mean ± 95% CI of the receiver operating characteristic area under the curve (AUC) is displayed as a bar graph in SVO and LAA AIS patients. The prediction models built with TI and II were found to be significantly better than the models built with RI both in SVO and LAA population. (A) the AUC of LR model with various combination of input in SVO and LAA AIS population for different outcome; (B) The differences between LR models of different input. AIS, acute ischemic stroke; AUC, area under curve; II, image index; LAA, large artery atherosclerosis; RI, regular index; SVO, small vessel occlusion; TI, total index.

In the LR models to predict outcomes of SVO‐AIS population, the LR model showed that the AUC of TI input (excellent outcome: 0.86 [0.78–0.94], good outcome: 0.88 [0.8–0.96]) was significantly better than that of RI input (excellent outcome: 0.66 [0.53–0.78], good outcome: 0.7 [0.58–0.83]) in comparison of prognostic predictive power. The results were similar in the LAA‐AIS population, and TI input had the best performance (excellent outcome: 0.73 [0.54–0.91], good outcome: 0.75 [0.59–0.91]). (Figure 1).

**FIGURE 2:**
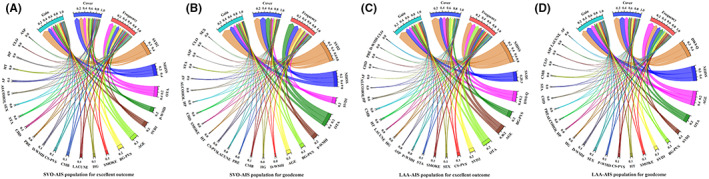
Important features selection analysis by GOA‐XGBoost algorithm for excellent outcome in SVO‐AIS population (A), good outcome in SVO‐AIS population (B), excellent outcome in LAA‐AIS population (C), and good outcome in LAA‐AIS population (D). AIS, acute ischemic stroke; ASP, aspirin; BG‐PVS, basal ganglia perivascular space; CHD, coronary heart disease; CLO, clopidogrel; CMB, cerebral microbleed; CS‐PVS, central semiovale perivascular space; DWI‐Q, diffusion weight image quantity; D‐WMH, deep white matter hyperintensity; GOA‐XGBoost, Grasshopper Optimization Algorithm eXtreme Gradient Boosting; HG, hyperglycemia; HP, hyperlipemia; HT, hypertension; LAA, large artery atherosclerosis; NIHSS, National Institute of Health Stroke Scale; OTA, onset‐to‐admission; PRE, premedication; P‐WMH, periventricular white matter hyperintensity; STA, statin; SVD1, total small vessel disease score; SVD2, modified total small vessel disease score; SVO, small vessel occlusion; AF, atrial fibrillation.

In the ranking of features based on GOA‐XGBoost algorithms, the specific indicators (gain, cover, and frequency values) of the importance of each index were shown in Tables S2–S5 and Figure 2. The modified total SVD score (Gain: 0.38, 0.28) plays an important role in predicting both excellent and good outcomes in SVO‐AIS population. However, in LAA‐AIS patients, the National Institute of Health Stroke Scale (NIHSS) (Gain: 0.32) was strongly correlated with excellent outcomes and diffusion weight image quantity (DWI‐Q) (Gain: 0.22) ranking first in predicting good outcomes.

**FIGURE 3:**
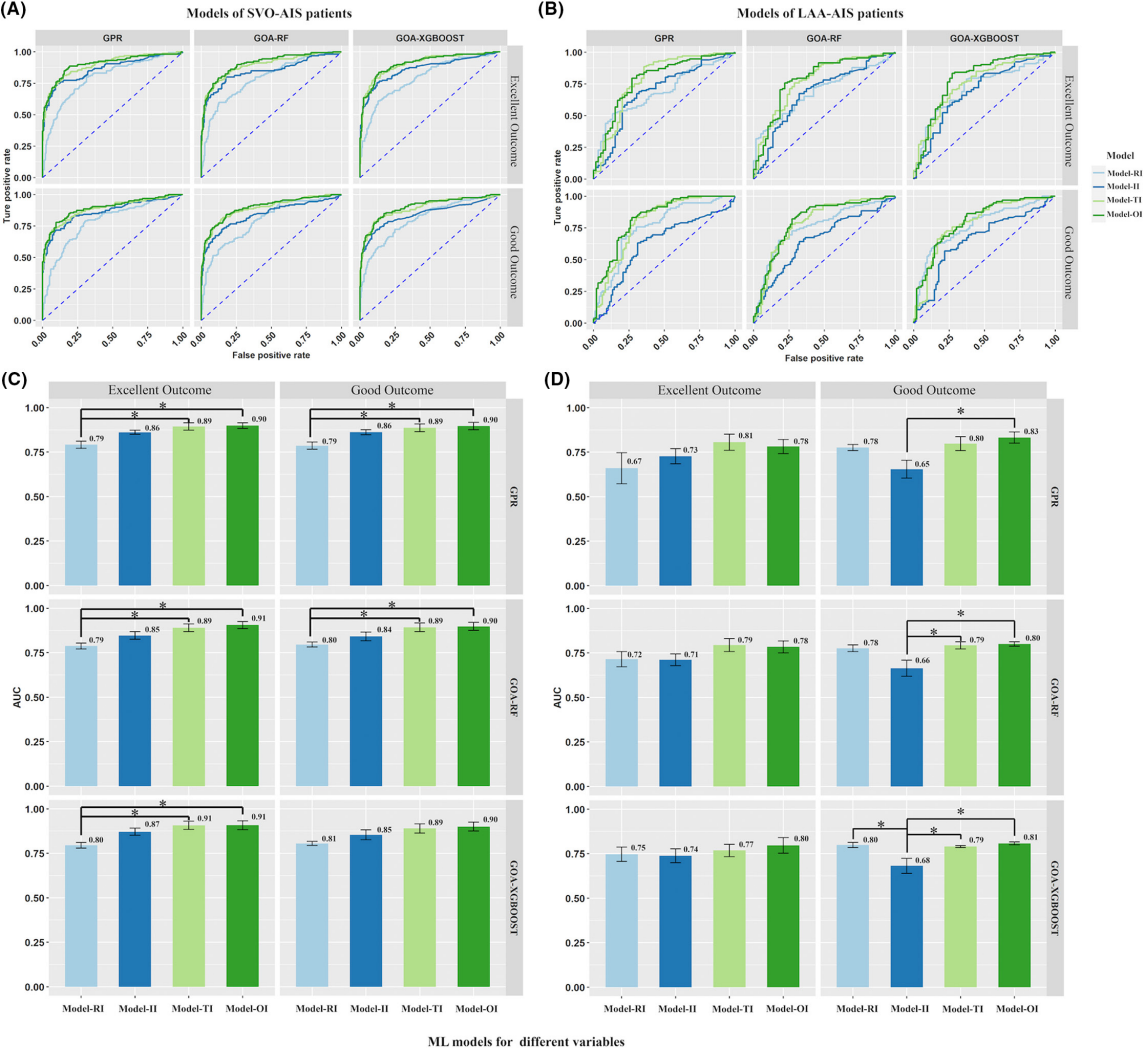
The OI input in different ML algorithms performed better than other inputs. In the ML models for good outcome, the models built with II alone had significantly lower predictive power for prognosis than the other input combinations. Mann–Whitney *U* test with Bonferroni correction was used. (A), (C) The AUC of ML models with various combination of input in SVO‐AIS population for excellent and good outcome; (B), (D) The AUC of ML models with various combination of input in LAA‐AIS population for excellent and good outcome. The mean ± 95% CI of the receiver operating characteristic area under the curve (AUC) is displayed as a bar graph. AIS, acute ischemic stroke; GOA‐RF, Grasshopper Optimization Algorithm Random Forest; GOA‐XGBoost, Grasshopper Optimization Algorithm eXtreme Gradient Boosting; GPR, Gaussian Process Regression; II, image index; LAA, large artery atherosclerosis; OI, optimal index; RI, regular index; SVO, small vessel occlusion; TI, total index.

In all 3 ML prediction models for SVO‐AIS population, models established by II input independently have favorable predictive power (GPR: excellent outcome: 0.86 [0.77–0.95], good outcome: 0.86 [0.77–0.96]; GOA‐RF: excellent outcome: 0.85 [0.75–0.94], good outcome: 0.84 [0.74–0.94]; GOA‐XGBoost: excellent outcome: 0.87 [0.79–0.96], good outcome: 0.85 [0.76–0.94]), although poorer than models built with OI input (GPR: excellent outcome: 0.90 [0.82–0.97], good outcome: 0.90 [0.82–0.97]; GOA‐RF: excellent outcome: 0.91 [0.84–0.97], good outcome: 0.90 [0.82–0.97]; GOA‐XGBoost: excellent outcome: 0.91 [0.84–0.97]), good outcome: 0.90 [0.83–0.97]), there were no significant statistical differences (Figure 3A,C). In contrast, the opposite results were found in the LAA‐AIS population, in the models built to predict good outcomes, the ability of GPR, GOA‐RF and GOA‐XGBoost ML algorithms with II input (GPR: 0.65 [0.47–0.83], GOA‐RF: 0.66 [0.49–0.84], GOA‐XGBoost: 0.68 [0.51–0.86]) was significantly lower than RI, TI, and OI input. (Figure 3B,D).

**FIGURE 4:**
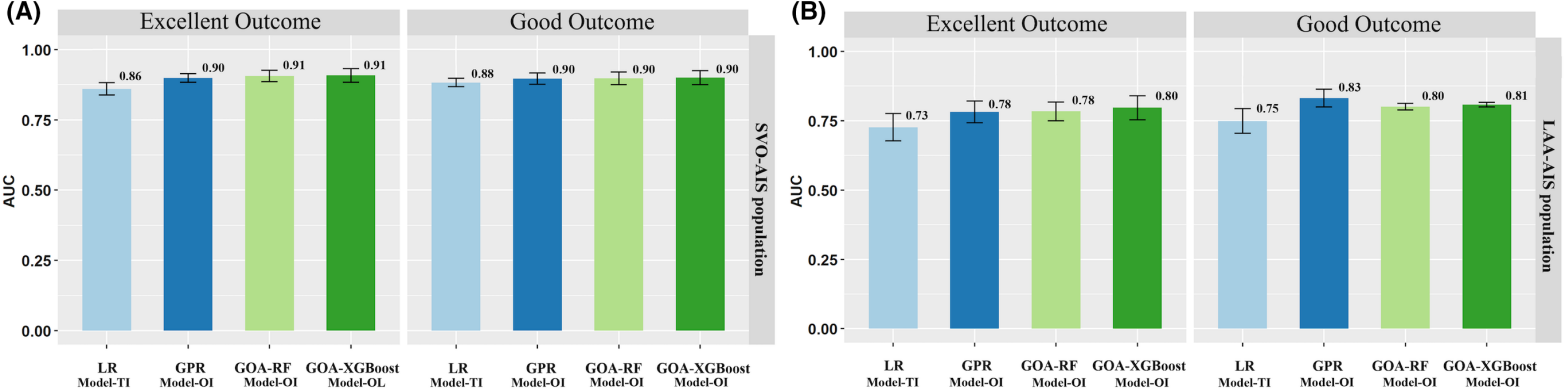
The mean ± 95% CI of the receiver operating characteristic area under the curve (AUC) is displayed as a bar graph on SVO and LAA AIS patients. There were no significant differences between logistic model (TI) and machine learning model (OI). (A) The performances of LR (TI) and ML (OI) models for different outcome in SVO‐AIS population; (B) The performances of LR (TI) and ML (OI) models for different outcome in LAA‐AIS population. SVO, small vessel occlusion; LAA, large artery atherosclerosis; LG, logistic; TI, total index; GPR, Gaussian Process Regression; GOA‐RF, Grasshopper Optimization Algorithm Random Forest; GOA‐XGBoost, Grasshopper Optimization Algorithm eXtreme Gradient Boosting.

Among all the prognostic models built with different inputs, the best‐performing TI input in the LR methods were compared with the OI input in the ML algorithms, and it was found that the GOA‐XGBoost models with the OI input has the best performance for SVO‐AIS population to predict excellent outcome. (0.91 [0.84–0.97]). (Figure 4) The detailed AUC values of all the LR and ML models are presented in Tables S6–S9.

## 4 DISCUSSION

To the best of our knowledge, this is the first study to assess the prognostic ability of SVD imaging markers on different subtypes of acute stroke using ML and LR methods. Most importantly, in SVO‐AIS patients, SVD‐related imaging markers alone precisely predict outcome, which is different from that achieved in the LAA‐AIS.

In the present study, SVD imaging markers differed among different subtypes of stroke patients. In SVO‐AIS patients, the modified total SVD score was the most effective feature in the prediction of excellent or good outcomes, and prediction models using SVD imaging markers alone could rapidly predict prognosis, reflecting the strong association between SVD burden and prognosis in SVO‐AIS patients. This phenomenon implies that SVO‐AIS always exhibits abnormalities in the penetrating or microscopic arteries, which is consistent with the state of microvascular function (“fertility” of the brain soil) represented by the SVD burden. However, in the LAA‐AIS population, the opposite results were observed, the models built in combination with SVD imaging markers and infarct volume performed worse than clinical indicators. The potentials explanatory mechanisms may be that LAA‐AIS is more probably associated with the dislodgement of large plaques which leads to blockage of large arteries, and therefore, patients with even larger infarct volumes still have better prognosis when good collateral is present. And in patients with stroke due to LAA, the impacts of large infarct volumes may overwhelm any added predictive value that concurrent SVD may provide. In addition, it has been shown that the brain tissue with high DWI signal still has the possibility for reversal in AIS patients with an earlier time window.^15^ Accordingly, the use of imaging markers alone (quantity of infarcts on DWI images combined with SVD imaging markers) cannot achieve satisfactory performance in prediction models for LAA‐AIS population. These results partially reflected the discrepancies in the etiology and intrinsic characteristics of the two different subtypes of AIS.

The mechanism by which SVD affects the prognosis of AIS patients is poorly understood. Previous studies have shown that the increased burden of WMH disrupts the microstructure of the brain as well as the network system, and the integrity of the brain network plays a substantial role in learning and neurological rehabilitation.^16^, ^17^ In the present study, periventricular WMH (P‐WMH) was more sensitive than deep WMH (D‐WMH) in predicting stroke outcomes. This phenomenon is analogous to the hypothesis of previous literature: P‐WMH rather than D‐WMH was preferentially associated with a decline in total cerebral blood flow and thus may be more vulnerable to hemodynamic disturbance and ischemia given the unstable blood supply of the periventricular watershed area.^18^, ^19^ Another often overlooked SVD imaging marker, PVS, has been reported to be related to an increased risk of recurrent ischemic stroke.^20^, ^21^, ^22^ However, we found that BG‐PVS had a more significant prognostic impact than CS‐PVS. This discrepancy can be explained by the anatomical and physiological mechanisms of PVS. CS‐PVS has only one layer of pia mater and represents spaces between the axon tracts. Regarding BG‐PVS, there are two layers of soft meningeal structures with small perforating arteries crossing it and connecting to the subarachnoid space, which are vulnerable to artery pulsations.^23^ Therefore, in 2017, Charidimou proposed that the increased number of PVS in the basal ganglia region is associated with risk factors for cerebrovascular atherosclerosis.^24^ In addition, chronic lacune and CMB were also found to be indictors of poor prognosis in previous studies.^2^, ^25^ However, in the present research, these two SVD imaging markers were not indispensable. Future larger multicenter studies are needed to investigate whether chronic lacune and CMB have a predictive effect on functional outcome in patients with AIS.

SVD features frequently occur in combination; therefore, it is necessary to quantify the total SVD burden to assess the cumulative effects of small‐vessel injury on the whole brain.^3^, ^26^ Therefore, preexisting total SVD burden may represent a marker of increased susceptibility of brain tissue to ischemia and other injuries, resulting in decreased neural reserve capacity of the brain.^27^ In this study, it was found that the modified 6‐point scale SVD score was the most significant independent variable in the ML prediction model due to its ability to better stratify WMH and CMB than the conventional 4‐point total SVD score, thus more elaborately representing the status of intracranial small vessels. Such SVD‐related brain injury may impair not only motor learning but also participation in rehabilitation and adherence to treatment guidelines, which leads to poor functional recovery.

The strengths of this study are the specific subdivision of acute stroke subtypes and the standardized collection of patient data. In addition, the best performance of the ML and LR models was compared due to extensive hyperparameter tuning and state‐of‐the‐art variable selection methods. The distinction between ML and LR methods mainly lies in the fact that ML can solve the problem of linearity of variables and overfitting of the model when the number of independent variables incorporated into the model is large.^28^ However, another clear difference among the various ML algorithms and LR exists in terms of model transparency. Logistic regression has the advantage of transparency at the level of individual variable coefficients given that odds ratios can be derived from these coefficients.^29^ In addition, the interpretability of ML models is poor, and the internal computing process is in a “black box” state. Moreover, it is difficult to explain the strengths and weaknesses of the models. The findings comparing ML and LR are controversial. In a study predicting the clinical outcomes of large vessel occlusion before MT, the RF model was significantly better than other ML algorithms and LR models.^29^ In contrast, a systematic review showed no performance benefit of ML over LR for clinical prediction models, and the accuracy of the different models was similar.^30^ This conclusion was similar to that noted in our work. The GOA‐XGBoost model performed the best, but no significant differences were found in the different models. In any case, ML evaluates the weights of variables more precisely. In traditional medical research, the variables were first evaluated using univariate logistic regression, and the important variables were simultaneously entered into the LR model.^31^, ^32^ This approach of variable selection has widely been used in the medical field; however, it is restricted to the problem of being weak to collinearity.

Several limitations to this study should be noted. First, the outcomes of AIS patients were considered dichotomous variables using clinically relevant cut points based on the literature and use of other prognostic indicators may be warranted in the future. Second, although this study basically covered most of the clinical features and combined subjective scores of SVD imaging markers to establish prognostic prediction models, objective metrics to quantify cerebral SVD could be used in the future, including the quantitative volume of WMH and PVS and diffusion tensor imaging to capture diffuse microstructural alterations, even in normal white matter. In addition, although our study lacks an external validation cohort, a fivefold cross‐validation approach is used to prevent model overfitting, and the performance of the model needs to be tested for its generalizability with various populations, such as those with different ethnicities and social backgrounds and undergoing different treatments. Finally, this was a retrospective study with a limited number of patients, and the model performance needs to be tested in a prospective study.

In conclusion, the modified SVD score plays an important role in SVO‐AIS patients, whereas in LAA‐AIS population, the NIHSS score and DWI quantity were essential in predicting outcomes, respectively. In the future clinical practices, for SVO‐AIS population, caregivers should pay more attention to the modified total SVD, P‐WMH, and BG‐PVS scores to identify those patients at higher risk for unfavorable functional outcomes and more clearly assessing the potential for functional recovery.

## AUTHOR CONTRIBUTION

X.L. initiated the project and the collaboration. X.L., L.C., and X.Y.W. designed study, interpreted results, and drafted manuscript. J.H.L., Z.H.M., X.Y.W., W.C., G.H.W., Q.L.N., X.L., Y.T.B., D.H., W.T.G., S.Y., X.B.B., Y.N.L., L.X.W., Q.D., T.Y.Z. collected and organized data. X.Y.W., C.H.D. analyzed data. X.Y.W. provided case selection and annotations. L.C. and X.L. provided critical comments and reviewed the manuscript.

## FUNDING INFORMATION

This work was supported by the National Natural Science Foundation of China (Nos. 81825012 and 81730048 to X.L., No 81901708 to J.H.L.).

## CONFLICTS OF INTEREST

Nothing to report.

## Supporting information


Appendix S1

## Data Availability

The data that support the findings of this study are available from the corresponding author upon reasonable request.
