# Supplementary material for: Small vessel disease burden predicts functional outcomes in patients with acute ischemic stroke using machine learning
Source: CNS Neurosci Ther. 2023 Jan 17;29(4):1024–33. doi: 10.1111/cns.14071 (PMC10018092; doi:10.1111/cns.14071)
Supplement: Supplementary file 1 — Appendix S1 [file CNS-29-1024-s001.docx]

**Supplement Table1:** Features and categories of different small vessel disease scores

| **Markers**  **Scores** | **Lacunes** | **Cerebral microbleeds (CMBs)** | **Perivascular spaces (PVS)** | **White matter hypertensity (WMH)** |
| --- | --- | --- | --- | --- |
| **The original SVD score**  **(0-4)** | Presence of lacunes, 1 point | Presence of CMB, 1 point | ＞10 BG-PVS, 1 point | PWMH (Fazekas=3) or DWMH (Fazekas=2-3), 1 point |
| **The modified SVD score**  **(0-6)** | Presence of lacunes, 1 point | 1-4 CMBs, 1 point ≥5 CMBs, 2 points | ＞20 BG-PVS, 1 point | Moderate WMH (total Fazekas=3-4), 1 point; Severe WMH (total Fazekas=5-6), 2 points |

SVD, small vessel disease; BG-PVS, basal ganglia perivascular space; CS-PVS, central semiovale perivascular space.

**Supplement Table 2:** Ranking of features based on GOA-XGBoost algorithm for excellent outcome in LAA-AIS population

| **Rank** | **Feature** | Gain | Cover | Frequency |
| --- | --- | --- | --- | --- |
| **1** | **NIHSS** | 0.318603 | 0.276471 | 0.163812 |
| **2** | **SVD1** | 0.139338 | 0.119913 | 0.065525 |
| **3** | **DWI-Q** | 0.09621 | 0.117264 | 0.137751 |
| **4** | **BG-PVS** | 0.093859 | 0.064725 | 0.058079 |
| **5** | **AGE** | 0.072409 | 0.11712 | 0.128071 |
| **6** | **OTA** | 0.068492 | 0.093618 | 0.131794 |
| **7** | **SVD2** | 0.05296 | 0.066664 | 0.046165 |
| **8** | **CS-PVS** | 0.030441 | 0.020146 | 0.04691 |
| **9** | **SEX** | 0.019729 | 0.011907 | 0.024572 |
| **10** | **SMOKE** | 0.01745 | 0.012873 | 0.032018 |
| **11** | **STA** | 0.014616 | 0.012133 | 0.023827 |
| **12** | **HT** | 0.014242 | 0.010819 | 0.021593 |
| **13** | **HP** | 0.010962 | 0.009151 | 0.01787 |
| **14** | **HG** | 0.009716 | 0.00922 | 0.020104 |
| **15** | **LACUNE** | 0.008963 | 0.005785 | 0.012658 |
| **16** | **P-WMH** | 0.008537 | 0.016524 | 0.015637 |
| **17** | **CMB** | 0.004981 | 0.019441 | 0.015637 |
| **18** | **ASP** | 0.004917 | 0.005618 | 0.011169 |
| **19** | **ALCOHOL** | 0.00425 | 0.002147 | 0.007446 |
| **20** | **CLO** | 0.003457 | 0.001991 | 0.005957 |
| **21** | **CHD** | 0.00234 | 0.002853 | 0.004468 |
| **22** | **PRE** | 0.002026 | 0.002334 | 0.004468 |
| **23** | **D-WMH** | 0.001399 | 0.000853 | 0.003723 |
| **24** | **AF** | 0.000103 | 0.00043 | 0.000745 |

GOA-XGBoost, Grasshopper Optimization Algorithm eXtreme Gradient Boosting; LAA, large artery atherosclerosis; AIS, acute ischemic stroke; NIHSS, National Institute of Health Stroke Scale; SVD1, total small vessel disease score; DWI-Q, diffusion weight image quantity; BG-PVS, basal ganglia perivascular space; OTA, onset-to-admission; SVD2, modified total small vessel disease score; CS-PVS, central semiovale perivascular space; STA, statin; HT, hypertension; HP, hyperlipemia; HG, hyperglycemia; P-WMH, periventricular white matter hyperintensity; CMB, cerebral microbleed; ASP, aspirin; CLO, clopidogrel; CHD, coronary heart disease; PRE, premedication; D-WMH, deep white matter hyperintensity; AF, atrial fibrillation.

**Supplement Table 3:** Ranking of features based on GOA-XGBoost algorithm for good outcome in LAA-AIS population

| **Rank** | **Feature** | Gain | Cover | Frequency |
| --- | --- | --- | --- | --- |
| **1** | **DWI-Q** | 0.216524 | 0.219511 | 0.160472 |
| **2** | **NIHSS** | 0.205975 | 0.196327 | 0.129794 |
| **3** | **AGE** | 0.152003 | 0.145168 | 0.215339 |
| **4** | **OTA** | 0.073925 | 0.089078 | 0.117994 |
| **5** | **SVD2** | 0.054871 | 0.049857 | 0.031858 |
| **6** | **BG-PVS** | 0.039002 | 0.034762 | 0.040708 |
| **7** | **SVD1** | 0.035679 | 0.0485 | 0.033628 |
| **8** | **SMOKE** | 0.027882 | 0.030025 | 0.030088 |
| **9** | **HT** | 0.026033 | 0.022297 | 0.030678 |
| **10** | **CS-PVS** | 0.025253 | 0.027809 | 0.021829 |
| **11** | **P-WMH** | 0.024009 | 0.025206 | 0.015929 |
| **12** | **SEX** | 0.022353 | 0.011329 | 0.044248 |
| **13** | **D-WMH** | 0.021566 | 0.025261 | 0.018879 |
| **14** | **HG** | 0.011814 | 0.00698 | 0.018879 |
| **15** | **HP** | 0.010353 | 0.006648 | 0.015339 |
| **16** | **ALCOHOL** | 0.009913 | 0.007922 | 0.014159 |
| **17** | **PRE** | 0.009376 | 0.011301 | 0.00708 |
| **18** | **CHD** | 0.007805 | 0.006371 | 0.011209 |
| **19** | **STA** | 0.006175 | 0.005817 | 0.00944 |
| **20** | **CMB** | 0.005966 | 0.009999 | 0.011209 |
| **21** | **CLO** | 0.004798 | 0.005817 | 0.0059 |
| **22** | **ASP** | 0.003589 | 0.006675 | 0.00708 |
| **23** | **LACUNE** | 0.003274 | 0.00421 | 0.00472 |
| **24** | **AF** | 0.001862 | 0.00313 | 0.00354 |

GOA-XGBoost, Grasshopper Optimization Algorithm eXtreme Gradient Boosting; LAA, large artery atherosclerosis; AIS, acute ischemic stroke; DWI-Q, diffusion weight image quantity; NIHSS, National Institute of Health Stroke Scale; OTA, onset-to-admission; SVD2, modified total small vessel disease score; BG-PVS, basal ganglia perivascular space; SVD1, total small vessel disease score; HT, hypertension; CS-PVS, central semiovale perivascular space; P-WMH, periventricular white matter hyperintensity; CLO, clopidogrel; D-WMH, deep white matter hyperintensity; PRE, premedication; ASP, aspirin; STA, statin; HG, hyperglycemia; CMB, cerebral microbleed; CHD, coronary heart disease; HP, hyperlipemia; AF, atrial fibrillation.

**Supplement Table 4:** Ranking of features based on GOA-XGBoost algorithm for excellent outcome in SVO-AIS population

| **Rank** | **Feature** | Gain | Cover | Frequency |
| --- | --- | --- | --- | --- |
| **1** | **SVD2** | 0.376942938 | 0.237927078 | 0.139963168 |
| **2** | **NIHSS** | 0.160626835 | 0.215729825 | 0.186003683 |
| **3** | **SVD1** | 0.114753713 | 0.061759589 | 0.044198895 |
| **4** | **OTA** | 0.101025415 | 0.17544744 | 0.216850829 |
| **5** | **P-WMH** | 0.073095217 | 0.07322183 | 0.063535912 |
| **6** | **BG-PVS** | 0.040321497 | 0.053902283 | 0.054327808 |
| **7** | **AGE** | 0.031388035 | 0.045544404 | 0.080110497 |
| **8** | **D-WMH** | 0.014478974 | 0.016130816 | 0.019337017 |
| **9** | **HG** | 0.011524265 | 0.011841734 | 0.018876611 |
| **10** | **CMB** | 0.010798853 | 0.019102451 | 0.020257827 |
| **11** | **PRE** | 0.009010645 | 0.010642309 | 0.01335175 |
| **12** | **LACUNE** | 0.009008876 | 0.013777341 | 0.01611418 |
| **13** | **CS-PVS** | 0.007964345 | 0.012883116 | 0.026243094 |
| **14** | **ALCOHOL** | 0.006224499 | 0.005084149 | 0.01427256 |
| **15** | **SMOKE** | 0.006063646 | 0.005311629 | 0.013812155 |
| **16** | **HT** | 0.005442156 | 0.004084147 | 0.011970534 |
| **17** | **CHD** | 0.005310132 | 0.013601391 | 0.01519337 |
| **18** | **HP** | 0.004776676 | 0.00387475 | 0.013812155 |
| **19** | **STA** | 0.00379313 | 0.006818579 | 0.010128913 |
| **20** | **AF** | 0.003541151 | 0.007884094 | 0.010128913 |
| **21** | **ASP** | 0.00167212 | 0.001828029 | 0.004143646 |
| **22** | **CLO** | 0.001276107 | 0.002023266 | 0.004143646 |
| **23** | **SEX** | 0.000960773 | 0.001579749 | 0.003222836 |

GOA-XGBoost, Grasshopper Optimization Algorithm eXtreme Gradient Boosting; SVO, small vessel occlusion; AIS, acute ischemic stroke; SVD2, modified total small vessel disease score; NIHSS, National Institute of Health Stroke Scale; SVD1, total small vessel disease score; OTA, onset-to-admission; P-WMH, periventricular white matter hyperintensity; BG-PVS, basal ganglia perivascular space; D-WMH, deep white matter hyperintensity; HG, hyperglycemia; CMB, cerebral microbleed; PRE, premedication; CS-PVS, central semiovale perivascular space; HT, hypertension; CHD, coronary heart disease; HP, hyperlipemia; STA, statin; AF, atrial fibrillation; ASP, aspirin; CLO, clopidogrel.

**Supplement Table 5:** Ranking of features based on GOA-XGBoost algorithm for good outcome in SVO-AIS population

| **Rank** | **Feature** | Gain | Cover | Frequency |
| --- | --- | --- | --- | --- |
| **1** | **SVD2** | 0.283935226 | 0.156056375 | 0.063141684 |
| **2** | **NIHSS** | 0.122178408 | 0.177839605 | 0.119096509 |
| **3** | **OTA** | 0.09304971 | 0.113549527 | 0.165297741 |
| **4** | **P-WMH** | 0.087335937 | 0.058540954 | 0.03798768 |
| **5** | **SVD1** | 0.084417321 | 0.071064746 | 0.050821355 |
| **6** | **AGE** | 0.062553071 | 0.067540064 | 0.126796715 |
| **7** | **BG-PVS** | 0.048693944 | 0.072311414 | 0.059034908 |
| **8** | **SMOKE** | 0.03077409 | 0.018631798 | 0.040041068 |
| **9** | **HG** | 0.02285501 | 0.019147332 | 0.033880903 |
| **10** | **LACUNE** | 0.021921233 | 0.041560839 | 0.028234086 |
| **11** | **CMB** | 0.021003013 | 0.036712201 | 0.039014374 |
| **12** | **CS-PVS** | 0.019305242 | 0.019081675 | 0.043634497 |
| **13** | **D-WMH** | 0.014454004 | 0.017694669 | 0.023100616 |
| **14** | **PRE** | 0.013830534 | 0.014278097 | 0.015913758 |
| **15** | **CHD** | 0.013354249 | 0.019337567 | 0.025154004 |
| **16** | **STA** | 0.01195459 | 0.014484013 | 0.020020534 |
| **17** | **SEX** | 0.009217552 | 0.008944556 | 0.020020534 |
| **18** | **ALCOHOL** | 0.008855315 | 0.009177106 | 0.020533881 |
| **19** | **AF** | 0.008111437 | 0.034378823 | 0.014887064 |
| **20** | **HT** | 0.007692157 | 0.006995422 | 0.014373717 |
| **21** | **HP** | 0.006957512 | 0.011303005 | 0.021560575 |
| **22** | **CLO** | 0.004795553 | 0.007367275 | 0.008726899 |
| **23** | **ASP** | 0.002754895 | 0.004002935 | 0.008726899 |

GOA-XGBoost, Grasshopper Optimization Algorithm eXtreme Gradient Boosting; SVO, small vessel occlusion; AIS, acute ischemic stroke; SVD2, modified total small vessel disease score; NIHSS, National Institute of Health Stroke Scale; onset-to-admission; P-WMH, periventricular white matter hyperintensity; SVD1, total small vessel disease score; BG-PVS, basal ganglia perivascular space; HG, hyperglycemia; CMB, cerebral microbleed; CS-PVS, central semiovale perivascular space; D-WMH, deep white matter hyperintensity; PRE, premedication; CHD, coronary heart disease; STA, statin; AF, atrial fibrillation; HT, hypertension; HP, hyperlipemia; CLO, clopidogrel; ASP, aspirin.

**Supplement Table 6:** Scores for the different models in LAA-AIS population to predict excellent outcome with 5-fold cross validation

| Model | Sensitivity | Specificity | Accuracy | AUC | AUC-low | AUC-up |
| --- | --- | --- | --- | --- | --- | --- |
| GPR (RI) | 39.3±29.5 | 70.6±4.9 | 54.9±14.1 | 0.67 | 0.49 | 0.83 |
| GPR (II) | 51.7±7.5 | 77.7±9 | 64.7±7.4 | 0.73 | 0.56 | 0.90 |
| GPR (TI) | 79.9±12.1 | 81.2±8.4 | 80.6±6.3 | 0.81 | 0.66 | 0.95 |
| GPR (OI) | 68.4±8.7 | 77.5±8.5 | 72.9±5.8 | 0.78 | 0.62 | 0.94 |
| GOA-RF (RI) | 53±17.9 | 73.6±7.6 | 63.3±11.2 | 0.72 | 0.55 | 0.88 |
| GOA-RF (II) | 48.9±6.7 | 74.3±6.4 | 36.0±6.4 | 0.71 | 0.53 | 0.89 |
| GOA-RF (TI) | 70.9±4.2 | 81.0±10.2 | 53.0±5.5 | 0.79 | 0.64 | 0.94 |
| GOA-RF (OI) | 69.6±10.1 | 81.2±7.6 | 75.4±7.3 | 0.78 | 0.62 | 0.95 |
| GOA-XGBoost (RI) | 52.9±9.4 | 76.4±9.5 | 64.6±7.1 | 0.75 | 0.57 | 0.92 |
| GOA-XGBoost (II) | 56.9±6.9 | 78.5±8.6 | 67.7±7 | 0.74 | 0.57 | 0.90 |
| GOA-XGBoost (TI) | 60.8±9.8 | 79.0±6 | 69.9±5.8 | 0.77 | 0.61 | 0.93 |
| GOA-XGBoost (OI) | 71±8 | 83±9.9 | 76.8±6.2 | 0.80 | 0.63 | 0.96 |
| Logistic (RI) | 33.1±8.1 | 71±10.3 | 52.0±7.4 | 0.53 | 0.34 | 0.72 |
| Logistic (II) | 44.1±9.0 | 75.6±10.4 | 59.9±7.7 | 0.66 | 0.49 | 0.83 |
| Logistic (TI) | 49.2±6.5 | 80.2±12.2 | 64.7±7 | 0.73 | 0.54 | 0.91 |

LAA, large artery atherosclerosis; AIS, acute ischemic stroke; AUC, area under curve; GPR, Gaussian Process Regression; RI, regular index; II, image index; TI, total index; OI, optimal index; GOA-RF, Grasshopper Optimization Algorithm random forest; GOA-XGBoost, Grasshopper Optimization Algorithm eXtreme Gradient Boosting.

**Supplement Table 7:** Scores for the different models in LAA-AIS population to predict good outcome with 5-fold cross validation

| Model | Sensitivity | Specificity | Accuracy | AUC | AUC-low | AUC-up |
| --- | --- | --- | --- | --- | --- | --- |
| GPR (RI) | 70.6±7.7 | 77.2±12.0 | 73.9±4.2 | 0.78 | 0.62 | 0.93 |
| GPR (II) | 65.1±12.3 | 65.4±16.5 | 65.3±9.6 | 0.65 | 0.47 | 0.83 |
| GPR (TI) | 71.5±11.5 | 72.6±11.7 | 72.1±6.8 | 0.80 | 0.65 | 0.94 |
| GPR (OI) | 72.7±9 | 72.9±9.3 | 72.8±5.9 | 0.83 | 0.70 | 0.96 |
| GOA-RF (RI) | 71.3±11.7 | 74.1±10.2 | 72.7±3.7 | 0.78 | 0.62 | 0.93 |
| GOA-RF (II) | 63.5±10 | 64.7±11.8 | 74.1±7 | 0.66 | 0.49 | 0.84 |
| GOA-RF (TI) | 75.4±10.7 | 73.0±7.8 | 74.2±4.8 | 0.79 | 0.64 | 0.94 |
| GOA-RF (OI) | 84.6±11.4 | 62.6±11.6 | 73.6±8.6 | 0.80 | 0.65 | 0.96 |
| GOA-XGBoost (RI) | 68.9±10.7 | 72.7±9.0 | 70.8±2.4 | 0.80 | 0.65 | 0.95 |
| GOA-XGBoost (II) | 63.4±9.3 | 64.8±16.3 | 64.1±8.6 | 0.68 | 0.51 | 0.86 |
| GOA-XGBoost (TI) | 73.9±9.3 | 75.2±9.1 | 74.5±1.5 | 0.79 | 0.63 | 0.95 |
| GOA-XGBoost (OI) | 71.0±7.6 | 74.1±11.7 | 72.5±3.2 | 0.81 | 0.66 | 0.95 |
| Logistic (RI) | 55±7.5 | 60.1±1.7 | 57.7±7.8 | 0.57 | 0.38 | 0.77 |
| Logistic (II) | 60.7±9.2 | 63.1±11.2 | 62±5.7 | 0.65 | 0.46 | 0.84 |
| Logistic (TI) | 63.3±7.1 | 71.7±15.7 | 67.2±7.1 | 0.75 | 0.59 | 0.91 |

LAA, large artery atherosclerosis; AIS, acute ischemic stroke; AUC, area under curve; GPR, Gaussian Process Regression; RI, regular index; II, image index; TI, total index; OI, optimal index; GOA-RF, Grasshopper Optimization Algorithm random forest; GOA-XGBoost, Grasshopper Optimization Algorithm eXtreme Gradient Boosting.

**Supplement Table 8:** Scores for the different models in SVO-AIS population to predict excellent outcome with 5-fold cross validation

| Model | Sensitivity | Specificity | Accuracy | AUC | AUC-low | AUC-up |
| --- | --- | --- | --- | --- | --- | --- |
| GPR (RI) | 74±2.7 | 74.7±7.8 | 74.3±4.4 | 0.79 | 0.69 | 0.90 |
| GPR (II) | 15.6±6.6 | 17.9±3.1 | 16.7±4.5 | 0.86 | 0.77 | 0.95 |
| GPR (TI) | 83±2.2 | 86.1±8.6 | 84.5±5.1 | 0.89 | 0.82 | 0.97 |
| GPR (OI) | 82.9±4.1 | 84.5±10.1 | 83.7±6.9 | 0.90 | 0.82 | 0.97 |
| GOA-RF (RI) | 78.1±2.6 | 74.2±6.9 | 76.2±4.5 | 0.79 | 0.68 | 0.89 |
| GOA-RF (II) | 81.5±1.8 | 78.7±6.6 | 80.1±4.0 | 0.85 | 0.75 | 0.94 |
| GOA-RF (TI) | 82.6±1.5 | 84.5±8 | 83.5±4.6 | 0.89 | 0.81 | 0.97 |
| GOA-RF (OI) | 84.3±3.5 | 85.3±9.3 | 84.8±6.1 | 0.91 | 0.84 | 0.97 |
| GOA-XGBoost (RI) | 77±4.4 | 74.9±8.1 | 76±4.6 | 0.80 | 0.69 | 0.90 |
| GOA-XGBoost (II) | 81.7±2.6 | 84.7±8.1 | 83.2±5.1 | 0.87 | 0.79 | 0.96 |
| GOA-XGBoost (TI) | 85.9±3.5 | 85.2±7.3 | 85.6±5.3 | 0.91 | 0.84 | 0.97 |
| GOA-XGBoost (OI) | 83.9±2.2 | 82.6±8.1 | 83.3±5.1 | 0.91 | 0.84 | 0.97 |
| Logistic (RI) | 67.7±3.7 | 62.2±12.3 | 65±7.6 | 0.66 | 0.53 | 0.78 |
| Logistic (II) | 75.3±1.7 | 80.3±7.3 | 77.8±3.6 | 0.81 | 0.71 | 0.91 |
| Logistic (TI) | 77.7±2.3 | 81.2±7.7 | 79.5±4.4 | 0.86 | 0.78 | 0.94 |

SVO, small vessel occlusion; AIS, acute ischemic stroke; AUC, area under curve; GPR, Gaussian Process Regression; RI, regular index; II, image index; TI, total index; OI, optimal index; GOA-RF, Grasshopper Optimization Algorithm random forest; GOA-XGBoost, Grasshopper Optimization Algorithm eXtreme Gradient Boosting.

**Supplement Table 9:** Scores for the different models in SVO-AIS population to predict good outcome with 5-fold cross validation

| Model | Sensitivity | Specificity | Accuracy | AUC | AUC-low | AUC-up |
| --- | --- | --- | --- | --- | --- | --- |
| GPR (RI) | 80.3±2.8 | 78.6±22 | 79.4±10 | 0.79 | 0.68 | 0.89 |
| GPR (II) | 90±3.9 | 80.2±11.8 | 84.9±7.7 | 0.86 | 0.77 | 0.95 |
| GPR (TI) | 90.2±3 | 84.4±10.6 | 87.3±6.5 | 0.89 | 0.81 | 0.97 |
| GPR (OI) | 90.2±2.5 | 85.1±9.4 | 87.7±5.7 | 0.90 | 0.82 | 0.97 |
| GOA-RF (RI) | 83.6±2.9 | 69.9±14.8 | 76.8±6.5 | 0.80 | 0.69 | 0.90 |
| GOA-RF (II) | 88.9±3.4 | 67.9±10.8 | 78.4±6.8 | 0.84 | 0.74 | 0.94 |
| GOA-RF (TI) | 90.8±3.9 | 80.3±10 | 85.6±6.6 | 0.89 | 0.82 | 0.97 |
| GOA-RF (OI) | 90.6±3.6 | 76.9±9.1 | 83.7±6 | 0.90 | 0.82 | 0.97 |
| GOA-XGBoost (RI) | 84.6±3.5 | 70.8±16.2 | 77.7±7.6 | 0.81 | 0.70 | 0.91 |
| GOA-XGBoost (II) | 91.1±3.9 | 78.2±12.1 | 84.6±7.8 | 0.85 | 0.76 | 0.94 |
| GOA-XGBoost (TI) | 90.3±2.9 | 75.4±8.5 | 82.9±5.4 | 0.89 | 0.81 | 0.97 |
| GOA-XGBoost (OI) | 90.6±3.2 | 77.4±10.3 | 84±6.6 | 0.90 | 0.83 | 0.97 |
| Logistic (RI) | 81.1±5.3 | 50.1±7.6 | 65.6±4.5 | 0.70 | 0.58 | 0.83 |
| Logistic (II) | 87.1±5 | 55.8±8.7 | 72±5.5 | 0.83 | 0.73 | 0.93 |
| Logistic (TI) | 88.7±4.7 | 66.7±4 | 77.9±3.3 | 0.88 | 0.80 | 0.96 |

SVO, small vessel occlusion; AIS, acute ischemic stroke; AUC, area under curve; GPR, Gaussian Process Regression; RI, regular index; II, image index; TI, total index; OI, optimal index; GOA-RF, Grasshopper Optimization Algorithm random forest; GOA-XGBoost, Grasshopper Optimization Algorithm eXtreme Gradient Boosting.

Supplement Figure 1:


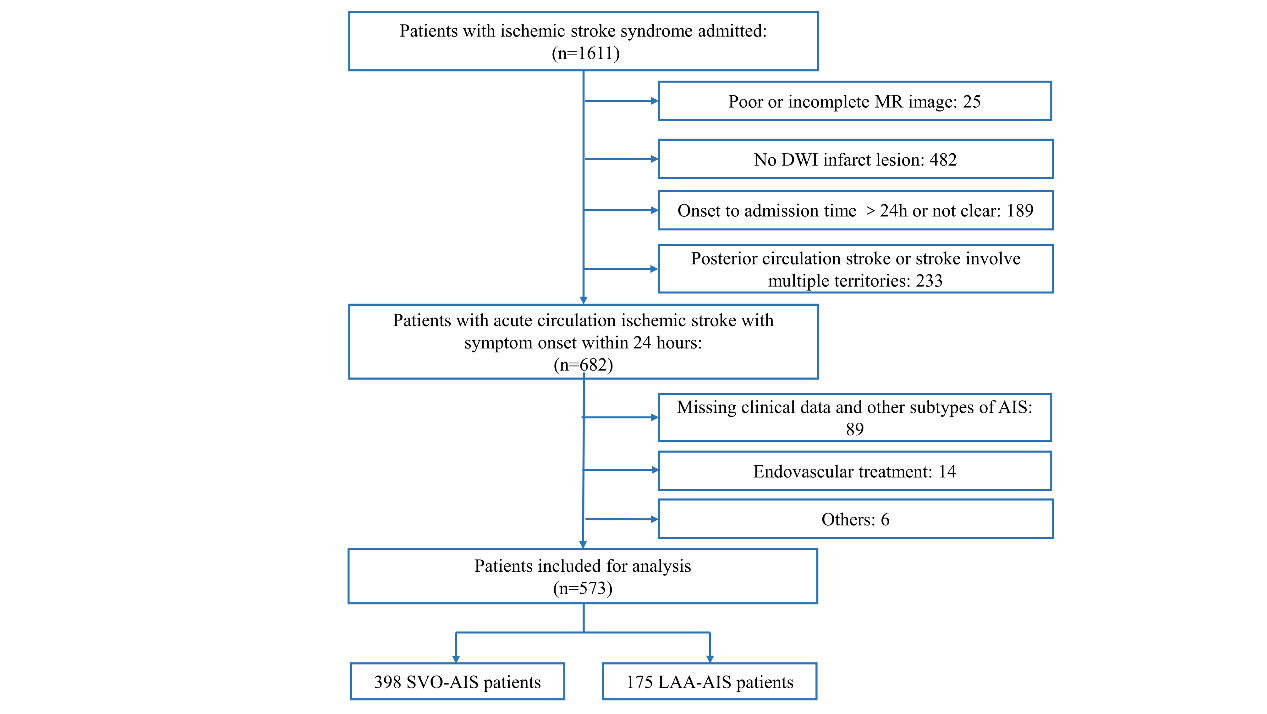


Patient selection. MR, magnetic resonance; AIS, acute ischemic stroke; LAA, large artery atherosclerosis; SVO, small vessel occlusion.
